# Differences in brain morphology of brown trout across stream, lake, and hatchery environments

**DOI:** 10.1002/ece3.8684

**Published:** 2022-03-08

**Authors:** Libor Závorka, J. Peter Koene, Tiffany A. Armstrong, Lena Fehlinger, Colin E. Adams

**Affiliations:** ^1^ WasserCluster Lunz – Inter‐University Centre for Aquatic Ecosystem Research Lunz am See Austria; ^2^ 3526 Institute of Biodiversity, Animal Health and Comparative Medicine College of Medical, Veterinary and Life Sciences University of Glasgow Glasgow UK; ^3^ 3526 Scottish Centre for Ecology and the Natural Environment (SCENE) University of Glasgow Glasgow UK

**Keywords:** animal cognition, hatchery environment, intraspecific variably, Omega‐3 long‐chain fatty acids, trophic interactions

## Abstract

It has been suggested that a trade‐off between cognitive capacity and developmental costs may drive brain size and morphology across fish species, but this pattern is less well explored at the intraspecific level. Physical habitat complexity has been proposed as a key selection pressure on cognitive capacity that shapes brain morphology of fishes. In this study, we compared brain morphology of brown trout, *Salmo trutta*, from stream, lake, and hatchery environments, which generally differ in physical complexity ranging from low habitat complexity in the hatchery to high habitat complexity in streams and intermediate complexity in lakes. We found that brain size, and the size of optic tectum and telencephalon differed across the three habitats, both being largest in lake fish with a tendency to be smaller in the stream compared to hatchery fish. Therefore, our findings do not support the hypothesis that in brown trout the volume of brain and its regions important for navigation and decision‐making increases in physically complex habitats. We suggest that the observed differences in brain size might be associated with diet quality and habitat‐specific behavioral adaptations rather than physical habitat complexity.

## Graphical abstract

The trade‐off between the information‐processing capacity and the developmental costs is suggested to drive brain morphology across fish species. We compared brain morphology of brown trout from three habitats differing in their physical complexity and quality of available diet. The overall brain size and size of telencephalon differed across the habitats, and the differences appeared to associate with diet quality and habitat‐specific behavioral adaptations rather than physical habitat complexity.

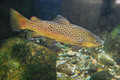

## 1 INTRODUCTION

The brain is the anatomical structure that limits the information‐processing capacity necessary for behavioral adaptation in vertebrate animals (Dicke & Roth, 2016; Kotrschal et al., 1998; Pike et al., 2018). It is suggested that brain size is driven by the trade‐off between the benefits provided by cognitive skills and the costs for its development and maintenance (Boogert et al., 2018; Morand‐Ferron & Quinn, 2015). Previous studies have shown that the brains of fishes often respond to selection pressure as modular organs, implying that only the volumes of specific regions controlling required cognitive skills under selection will increase, while the volumes of brain regions that are not used will shrink, possibly as an energy‐saving adaptation (Fong et al., 2021; Noreikiene et al., 2015; Pike et al., 2018). This mosaic evolution model, which has first been first described in mammals (Barton & Harvey, 2000; De Winter & Oxnard, 2001), is not mutually exclusive with partial developmental constraints of overall brain size, where developmental processes synchronously regulate the whole‐brain‐size change as a single unit (e.g., Finlay & Darlington, 1995; Noreikiene et al., 2015). The observed correlation of brain size and morphology with physical habitat complexity has led to the assumption that physical habitat complexity is one of the key drivers shaping brain morphology and size across fish species (Kotrschal et al., 1998; Pollen et al., 2007; Shumway, 2008). More complex habitats appear to select for larger brains and, particularly, for the larger brain regions that facilitate spatial navigation and complex decision making (i.e., telencephalon), perception of visual cues (i.e., optic tectum), and motor coordination (i.e., cerebellum) (Kotrschal et al., 1998; Pollen et al., 2007). These differences in brain morphology among fishes can be evolutionary (Kotrschal et al., 1998; Pollen et al., 2007) or plastic (e.g., Näslund et al., 2012; Triki et al., 2019) and also driven by evo‐devo processes (Sylvester et al., 2010). However, the association between physical habitat complexity and brain morphology in wild fishes has been much less studied at the intraspecific than at the interspecific level.

A study on three‐spined sticklebacks, *Gasterosteus aculeatus*, has shown differences in brain morphology among populations from lake and stream habitats (Ahmed et al., 2017), but these differences were not always consistent with the prediction that individuals from the more physically complex stream habitat have larger telencephala than individuals from the less complex lake habitat (Ahmed et al., 2017). Lake fishes can also experience high habitat complexity in lakes with a well‐developed littoral zone, but fishes in lakes with a simple shoreline should generally experience lower physical habitat complexity than stream‐dwelling conspecifics (Ahmed et al., 2017; Park & Bell, 2010). Therefore, what drives the differences in brain morphology among lake‐ and stream‐dwelling populations of fishes remains an open question. Overlooked in this context is the potential effect of diet quality on brain development. Brain development is inherently linked to dietary supply of energy and nutrients, particularly of omega‐3 long‐chain polyunsaturated fatty acids (n‐3 LC‐PUFA) (Pilecky et al., 2021). The availability of these nutrients differs across ecosystems, and they are more available to fish in lake habitats than in streams, because stream habitats receive a relatively higher input of terrestrial organic matter that is poor in n‐3 LC‐PUFA (Guo et al., 2021; Heissenberger et al., 2010; Hixson et al., 2015). High amounts of dietary n‐3 LC‐PUFA are also typical for the diet of hatchery‐reared fish (Heissenberger et al., 2010; Tocher, 2015), which are also raised in habitats with extremely low physical complexity (Kihslinger et al., 2006; Näslund et al., 2012). Therefore, a comparison of brain morphology across individuals from stream, lake, and hatchery environments can provide an insight into intraspecific differences in brain size and morphology associated with habitat quality in freshwater fishes.

Brown trout, *Salmo trutta* L., is a good model species for such a comparative study, because genetically and phenotypically different populations of brown trout occur in both lake and stream habitats (Jonsson & Jonsson, 2011). A previous study has shown differences in brain morphology of anadromous and stream resident brown trout; it was suggested that these are driven by differences in sex‐specific reproduction strategies rather than by physical habitat complexity (Kolm et al., 2009). Brown trout, like other salmonids, are also often reared in extremely simple hatchery environments (Kihslinger et al., 2006; Näslund et al., 2012). Some evidence suggests that the brains of hatchery‐reared individuals differ from the brains of conspecifics living in the wild due to a plastic response to the simplicity of the environment in which they developed (Kihslinger et al., 2006; Näslund et al., 2012), and due to genetic changes and artificial selection pressure on the hatchery strains (e.g., Kotrschal et al., 2012). In this study, we aim to compare brain morphology of brown trout from stream, lake, and hatchery environments in order to test how brain morphology varies across environments that differ in physical habitat complexity and quality of available diet. We predict the average size of trout brains should be largest in streams, smaller in lakes and smallest in hatchery fish if physical habitat complexity is the dominant driver, but largest in hatchery and smallest in streams if diet quality is the dominant diver of the brain size.

## 2 MATERIALS AND METHODS

### 2.1 Fish sampling and data collection

In January 2019 brown trout were captured in Scotland, UK, from Loch Sloy (56.2632175°N, 4.7707667°W; *N* = 14; FL = 261 ± 52 mm mean±SD) and Carron Valley Reservoir (56.0338314°N, 4.1057406°W; *N* = 14; FL = 227 ± 37 mm) using 30 m × 1.5 m single‐mesh (38 mm) benthic gill nets. Three nets were deployed in late afternoon and retrieved the following morning. Loch Sloy has a surface area of 1.33 km^2^ and a mean depth of 25.5 m. Carron Valley Reservoir has a surface area 3.76 km^2^ and a mean depth of 9.6 m. The fish community in Loch Sloy is composed of brown trout, European whitefish *Coregonus lavaretus*, and European eel *Anguilla anguilla*. Carron Valley Reservoir contains the same fish species as Loch Sloy complemented by rainbow trout *Oncorhynchus mykiss*, three‐spined stickleback, and Eurasian perch *Perca fluviatilis*. In October 2019, brown trout were captured by electrofishing (e‐fish, UK) in small tributaries of Loch Lomond, Scotland, UK (56.0470272°N, 4.5504428°W): the Ross Burn (*N* = 12, FL = 153 ± 16 mm) and Wood Burn (*N* = 3, FL = 165±42 mm). The Ross Burn has a mean discharge of 0.3 m^3^.s^−1^ and a length of 2.3 km and Wood Burn has a mean discharge of 1.1 m^3^.s^−1^ and a length of 3.1 km. Fish communities in Ross Burn and Wood Burn are the same, and composed of brown trout, brook lamprey *Lampetra planeri*, European eel, and occasional Atlantic salmon *Salmo salar* and European minnow *Phoxinus phoxinus*. The composition of fish communities suggests that the potential predation pressure did not differ across sampled lakes and streams with large trout and eel being the only potential fish predators of juvenile brown trout. Finally, in January 2020, hatchery young‐of‐year brown trout (Ae Fishery, Moffat, UK) were transported to the Scottish Centre for Ecology and the Natural Environment (SCENE). Hatchery trout were held in unadorned 120‐L cylindrical tanks at a density of 20 fish per tank. Tanks were fed individually with water on a flow‐through system directly from Loch Lomond at natural temperatures (low of ~3° C in winter, to high of ~18° C in summer). Inflowing water of ~100 L per hour was directed to provide a current and an air stone was added to each tank. Lighting simulated ambient sunlight at the latitude of the facility (~56° N). Hatchery trout were fed daily to satiation on commercial salmon pellets containing 31.3% of fish oil (Ewos Ltd., UK). These commercial pellets are a standard diet of hatchery trout and contain a high amount of n‐3 LC‐PUFA (Heissenberger et al., 2010). Hatchery fish were held under these conditions until June 2020, when brain samples of randomly selected individuals were extracted (*N* = 15, FL = 178 ± 22 mm). All fish were euthanized with an overdose of benzocaine. The heads of fish were removed and fixed in 4% buffered (pH 6.9) paraformaldehyde solution. Brains were then dissected out by opening the skull along the anteroposterior axis and removing muscle tissue and bones around the brain until the brain could be lifted from the skull. Dissected brains were stored in 4% buffered paraformaldehyde until further procedures were conducted. Brains were photographed with a Canon EOS 1300D DSLR camera with an EF‐S18–55 III lens (Canon) and 13‐ and 31‐mm extension tubes designed for Canon DSLRs (Xit Inc.). For each dissected brain sample, an image was taken from dorsal, left lateral, and ventral views (Appendix S1). Each brain was measured from dorsal, ventral, and lateral perspectives to calculate total volume and the volumes of the cerebellum, optic tectum, telencephalon, olfactory bulb, and hypothalamus. Measurements of total brain length, width and depth were taken independently of the measurements of brain regions for the calculation of total volume (Appendix S1). Measurements were completed using ImageJ 1.48 (Schneider et al., 2021) and used to calculate volumes using the formulas outlined by Pollen et al. (2007).

We performed geometric morphometric analysis of body shape to confirm the predominant habitat use of wild caught individuals (see Appendix S2). This analysis indicated clear differences in body shape of individuals caught in lake and stream habitats (Procrustes ANOVA: *F*_2,64_ = 11.6, *p* = .001), which suggests that the presence of individuals in the sampled habitat (i.e., lake or stream) was not coincidental, but corresponds to their long‐term habitat preference.

### 2.2 Statistical analysis

All analyses were conducted in R v.4.0.2 (R Core Team, 2020). We tested the effect of habitat on overall brain volume using a linear model with habitat (a categorical variable: lake, stream, hatchery), fork length, and their interaction. The effect of habitat on brain regions was tested by linear models with habitat and total brain volume minus the volume of the region of interest (Pike et al., 2018), and their interaction. All sampled individuals have been used for this analysis (*N* = 45). Variables in all models were log‐transformed. Nonsignificant interaction terms were removed from models. The significance of the explanatory variables was evaluated using ANOVA tables using Type II and III sums of squares for models without, and with, the interaction term, respectively. Deviations from the assumptions of the models were diagnosed from the distribution of model residuals and the association of fitted and residual values. The assumptions of the models were met in all cases. Differences between categories of habitat were analyzed using Tukey's HSD post hoc test.

Since the body‐size distribution of individuals was uneven across the three habitats (*F*_2,52_ = 31.48, *p* < .001, model *R* ^2^adj. = 0.530), with lake fish being significantly larger on average than stream (post hoc *p* < .001) and hatchery reared fish (post hoc *p* < .001), the above‐described models were rerun using a common‐body‐size window where fish from the three environments overlapped (i.e., fork length 150–215 mm). This reduced our sample size to 29 individuals (hatchery *N* = 14, lake *N* = 8, stream *N* = 7) but allowed us more robust control over the potential allometric differences in brain development in the three environments (Appendix S3).

## 3 RESULTS

**FIGURE 1:**
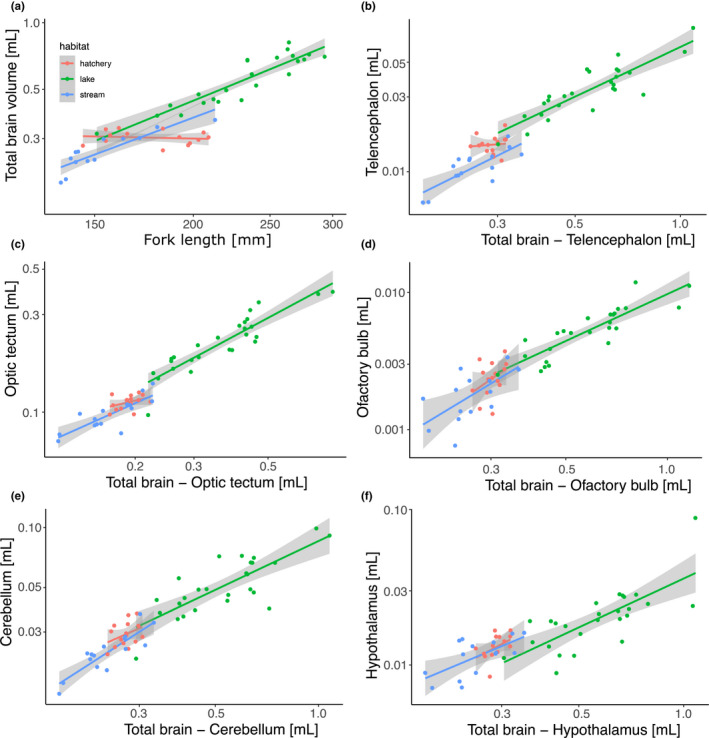
The log‐log scale relationship between (a) overall brain volume and fork length (i.e., encephalization), and between overall brain volume and volume of (b) the telencephalon, (c) optic tectum, (d) olfactory bulb, (e) cerebellum, and (f) hypothalamus

Overall brain volume was affected by the interaction between habitat and the fork length (FL) of individuals (*F*_2,49_ = 22.61, *p* < .001, model *R* ^2^adj. = 0.947). The significant interaction indicates that while there was a positive link between the overall brain volume and FL in wild populations, that is, lake and stream habitat (*F*_1,37_ = 214.28, *p* < .001, model *R* ^2^adj. = 0.947, Table 1), hatchery fish showed no association between brain volume and FL (*F*_1,13_ = 0.2431, *p* = .6302, model *R* ^2^adj. = 0.000, Figure 1a, Table 1). Relative brain volume was larger in fish from the lake than from the stream habitat (*F*_1,37_ = 5.4795, *p* = .0247). The telencephalon volume increased with increasing volume of the whole brain (*F*_1,52_ = 109.49, *p* < .001, model *R* ^2^adj. = 0.912, Figure 1b); however; telencephalon volume also differed significantly between fish from different habitats (*F*_2,52_ = 8.164, *p* = .001). Specifically, the telencephala of stream‐dwelling trout were smaller than those of trout from both lake and hatchery environments (post hoc *p* < .006), but the telencephala of lake and hatchery trout did not differ from each other (post hoc *p* = .252). The volume of the optic tectum increased with the increasing volume of the whole brain (*F*_1,52_ = 175.51, *p* < .001, model *R* ^2^adj. = 0.931, Figure 1c; Table 1), and optic tectum volume also differed significantly between fish from different habitats (*F*_2,52_ = 3.723, *p* = .031). The optic tecta of lake‐dwelling trout were significantly larger than those of stream trout (post hoc *p* = .027) and tended to be larger than the optic tecta of hatchery trout (post hoc *p* = .054); but stream and hatchery trout did not differ from each other (post hoc *p* = .733). However, the difference in volume of optic tecta among the habitats was not significant when considering only the subset of individuals from the common‐size window (Appendix S3). The volumes of the olfactory bulb, cerebellum, and hypothalamus increased with increasing volume of the whole brain (olfactory bulb: *F*_1,52_ = 61.42, *p* < .001, model *R* ^2^adj. = 0.808, Figure 1d; cerebellum: *F*_1,52_ = 80.70, *p* < .001, model *R* ^2^adj. = 0.847, Figure 1e; hypothalamus: *F*_1,52_ = 51.65, *p* < .001, model *R* ^2^adj. = 0.651, Figure 1f, Table 1), but volumes of these brain regions did not differ among individuals from different habitats (olfactory bulb: *F*_2,52_ = 0.639, *p* = .532; cerebellum: *F*_2,52_ = 1.421, *p* = .251; hypothalamus: *F*_2,52_ = 2.403, *p* = .100). In addition, considering only the subset of individuals from the common‐size window, the volume of hypothalamus was not significantly related to the overall brain volume (Appendix S3). All other results reported here were qualitatively similar for the analysis of both the full sample size and the subset of individuals from the common‐size window (Appendix S3).

**TABLE 1:** D.f., Intercept, Slope, p‐value, and *R* ^2^_adj_ of linear models between FL and total brain volume, and (total brain volume – brain region volume) and the brain region volume run separately for each habitat. The linear models are based on log‐transformed variables and correspond to the curves fitted in the Figure 1

Habitat	Brain region	*df*	Intercept	Slope	*p*‐value	*R* ^2^_adj_
Hatchery	Total brain	1;13	−0.801	−0.076	.630	0.000
	Telencephalon	1;13	−3.990	0.178	.737	0.000
	Optic tectum	1;13	−1.551	0.385	.136	0.099
	Olfactory bulb	1;13	−4.006	1.702	.139	0.096
	Cerebellum	1;13	−2.548	0.759	.159	0.081
	Hypothalamus	1;13	−2.861	1.189	.096	0.000
Stream	Total brain	1;13	−7.720	1.269	<.001	0.752
	Telencephalon	1;13	−3.037	1.106	<.001	0.590
	Optic tectum	1;13	−1.026	0.730	<.001	0.662
	Olfactory bulb	1;13	−4.333	1.490	.001	0.367
	Cerebellum	1;13	−2.168	1.106	<.001	0.736
	Hypothalamus	1;13	−3.188	0.943	.001	0.367
Lake	Total brain	1;23	−9.041	1.553	<.001	0.871
	Telencephalon	1;24	−2.772	1.053	<.001	0.746
	Optic tectum	1;24	−0.633	0.876	<.001	0.813
	Olfactory bulb	1;24	−4.626	1.136	<.001	0.734
	Cerebellum	1;24	−2.456	0.810	<.001	0.607
	Hypothalamus	1;24	−3.326	1.043	<.001	0.528

## 4 DISCUSSION

The relative brain volume, that is, after controlling for fish FL, was larger in lake habitats compared to streams. While volumes of all brain regions changed in close correlation to the overall brain volume, telencephalon volume was, when compared to the rest of the brain, disproportionately larger in lake than in stream‐dwelling trout. In addition, the telencephala of stream trout were smaller than the telencephala of hatchery trout. Similarly, to the study of Ahmed et al. (2017) on wild populations of stickleback, our findings do not support the hypothesis that in brown trout, the volumes of brain and its regions important for navigation and decision‐making, increase in the physically complex stream habitat, compared to the simpler lake and hatchery habitats.

We posit that the discrepancy between theoretical predictions and our findings can possibly be explained by two main mutually nonexclusive factors. First is the nonlinear association between physical habitat complexity and selection for larger brains, particularly for larger telencephala (Boogert et al., 2018). This explanation assumes that habitat complexity beyond a certain threshold may favor simple behavioral strategies to operate effectively in those complex environments. This is because their success probability is comparable to more complicated and cognitively demanding behaviors requiring costly investment in brain development (Morand‐Ferron & Quinn, 2015). Therefore, the high complexity of the rapidly changing stream habitat may favor simpler behavioral strategies than the less complex lake habitat. Stationary behavior and a sit‐and‐wait foraging strategy of stream‐dwelling trout (Jonsson & Jonsson, 2011) is an example of such simple behavioral adaptations to the complex stream habitat. In contrast, alternation in foraging between pelagic and littoral zones and more common piscivory among lake‐dwelling brown trout (Sánchez‐Hernández, 2020) may induce selection for large telencephala, and possibly also optic tecta, due to the need for visual‐cue processing and relatively complex navigation and decision‐making skills that they require (Edmunds et al., 2016).

A second potential explanation for differences in brain size between the habitats could be differences in available diet quality, which limit the nutrient supply for brain development. Lake‐dwelling trout feeding on zooplankton or other fish acquire more n‐3 LC‐PUFA than stream‐dwelling trout, which rely on a mix of macro‐zoobenthos and n‐3 LC‐PUFA‐poor terrestrial insects (Evangelista et al., 2014; Heissenberger et al., 2010; O'Grady, 1983; Sánchez‐Hernández & Cobo, 2016). Previous laboratory studies that have shown that the availability of dietary n‐3 LC‐PUFA has a positive effect on overall brain size (Lund et al., 2012) and on the size of optic tecta of fishes (Ishizaki et al., 2001). Therefore, the higher dietary intake of these nutrients may facilitate brain‐size development in lake‐dwelling brown trout, compared to their conspecifics from stream habitats. An extremely n‐3 LC‐PUFA‐rich diet is also typical for hatchery‐reared fish (Heissenberger et al., 2010; Tocher, 2015). This high‐quality diet may loosen the selection trade‐off between the cost of brain development and benefits of high cognitive capacity that is assumed to shape brain morphology in wild animals (Boogert et al., 2018; Morand‐Ferron & Quinn, 2015). Thus, a high‐quality diet, which enables rapid brain growth in hatchery fish, might explain the findings of this and other studies (Kotrschal et al., 2012; Näslund et al., 2012) that hatchery salmonids can have similar or larger brain and telencephalon volumes than stream‐dwelling salmonids, despite the low physical complexity of a hatchery environment. The relative brain size (i.e., encephalization) of hatchery trout was, in our study, difficult to compare with the wild fish due to the difference in the allometric relationship between the FL and brain size in hatchery and wild individuals. It appears that hatchery trout of smaller body size had relatively larger brains than wild conspecifics, but larger hatchery trout had relatively smaller brains than wild conspecifics. The slope of the relationship between FL and brain volume in the hatchery fish was much shallower than for the wild fish, even when we considered the common‐body‐size window where trout from all three habitats overlapped. Had we examined a sufficiently large range of body sizes in hatchery fish, we would likely eventually find a positive association of fork length and brain volume, but it should be noted that the hatchery fish, unlike the wild fish, typically grow at a similar rate, and thus they often have much lower within‐cohort variation in body sizes than wild fish. Therefore, our sample in this respect adequately represents the reality of the size distributions in the sampled populations. The lack of positive correlation between the brain size and FL shown here in hatchery individuals is unusual for wild fishes, where body size is a strong predictor of brain volume (e.g., Triki et al., 2021). Previous studies on the evolution of encephalization in mammals have suggested that relative brain size depends on selection pressure on the size of the brain as well as on the overall body size (Smaers et al., 2012). Therefore, high‐quality diet and selection for fast body growth in hatchery fish could partially uncouple the developmental link between the brain and the rest of the body (Kotrschal et al., 2012; Smaers et al., 2012).

Other factors that have been shown to influence brain size and morphology in fishes and were not explicitly considered in our comparative study include predation pressure (Burns & Rodd, 2008; Gonda et al., 2012; Kotrschal et al., 2017), sex (Kolm et al., 2009; Näslund, 2018), and ontogeny (Abrahao et al., 2021; Lisney et al., 2007; Lisney & Collin, 2006). Potential predators, such as eel and large trout, were present in all sampled wild populations (see methods section), and thus were unlikely to explain differences between the lake and stream habitats. The proportion of males and females in brown trout populations is generally similar (e.g., Baglinière et al., 1989) and thus should not differ between the groups compared in this study, although some studies suggest that female brown trout are more common in pelagic lake habitats (e.g., Jonsson, 1989). It has been shown that relative size of telencephala in wild fish can vary across seasons due changes in reproduction and habitat use (McCallum et al., 2014; Versteeg et al., 2021). However, we collected samples of wild brown trout in October and January, which is the period immediately before and after the peak of the spawning season of brown trout in this region (Campbell, 1977), so this seasonal variation should not affect our results. There were clearly ontogenetic differences between the compared groups, as hatchery trout were young‐of‐year, while the sizes of all wild trout corresponded to ages between 2 and 4 years (Maitland & Campbell, 1992). Lake‐dwelling trout were larger than stream trout, and thus some of the lake individuals could have been older than others in the sample, but these differences could also stem from differences in growth rates in lake and stream trout (Jonsson & Jonsson, 2011; Sánchez‐Hernández, 2020). The differences in growth rates among the environments compared in this study make it impossible to collect individuals of the same body size and age, and thus confounding of these two factors is an inherent shortcoming of any such comparative study. Brains of larger individuals become dominated by the forebrain, including the telencephalon (Finlay & Darlington, 1995), which suggests that the relatively larger telencephala of larger trout from the lakes compared to the smaller trout from the streams could be an artifact of ontogenetic development. However, when we considered only individuals from the common‐body‐size window, we still found that lake‐dwelling trout had significantly larger overall brain size and relatively larger telencephala than stream‐dwelling trout. Therefore, ontogenetic differences alone are unlikely to explain this variation in brain size and morphology. However, studies testing individuals across a broader range of habitats, body sizes, and ontogenetic stages are needed to confirm these patterns.

In conclusion, our study provides an example of among‐population variability in brain size and morphology, which is a topic still widely understudied in the wild. We suggest that, besides the cognitive demands of the environment (e.g., habitat complexity), future studies should also consider the availability of dietary essential fatty acids as a possible key driver of brain evolution and development in wild fishes.

## CONFLICT OF INTEREST

Authors have no conflict of interests to declare.

## AUTHOR CONTRIBUTIONS

**Libor Závorka:** Conceptualization (lead); Data curation (lead); Formal analysis (lead); Funding acquisition (lead); Methodology (lead); Project administration (lead); Resources (equal); Visualization (lead); Writing – original draft (lead). **J. Peter Koene:** Conceptualization (supporting); Investigation (equal); Methodology (equal); Validation (equal); Visualization (equal); Writing – review & editing (equal). **Tiffany A. Armstrong:** Data curation (equal); Methodology (equal); Resources (equal); Software (equal); Validation (equal); Writing – review & editing (equal). **Lena Fehlinger:** Investigation (equal); Validation (equal); Writing – review & editing (equal). **Colin E. Adams:** Conceptualization (equal); Funding acquisition (equal); Investigation (equal); Methodology (equal); Resources (equal); Writing – review & editing (lead).

## Supporting information

Supplementary Material

## Data Availability

Data are archived at figshare.com (https://doi.org/10.6084/m9.figshare.19152779).
