# Supplementary material for: Differences in brain morphology of brown trout across stream, lake, and hatchery environments
Source: Ecol Evol. 2022 Mar 8;12(3):e8684. doi: 10.1002/ece3.8684 (PMC8902666; doi:10.1002/ece3.8684)
Supplement: Supplementary file 1 — Supplementary Material [file ECE3-12-e8684-s001.docx]

***Appendix 1 – Photographs of trout brains across the three environments***

***
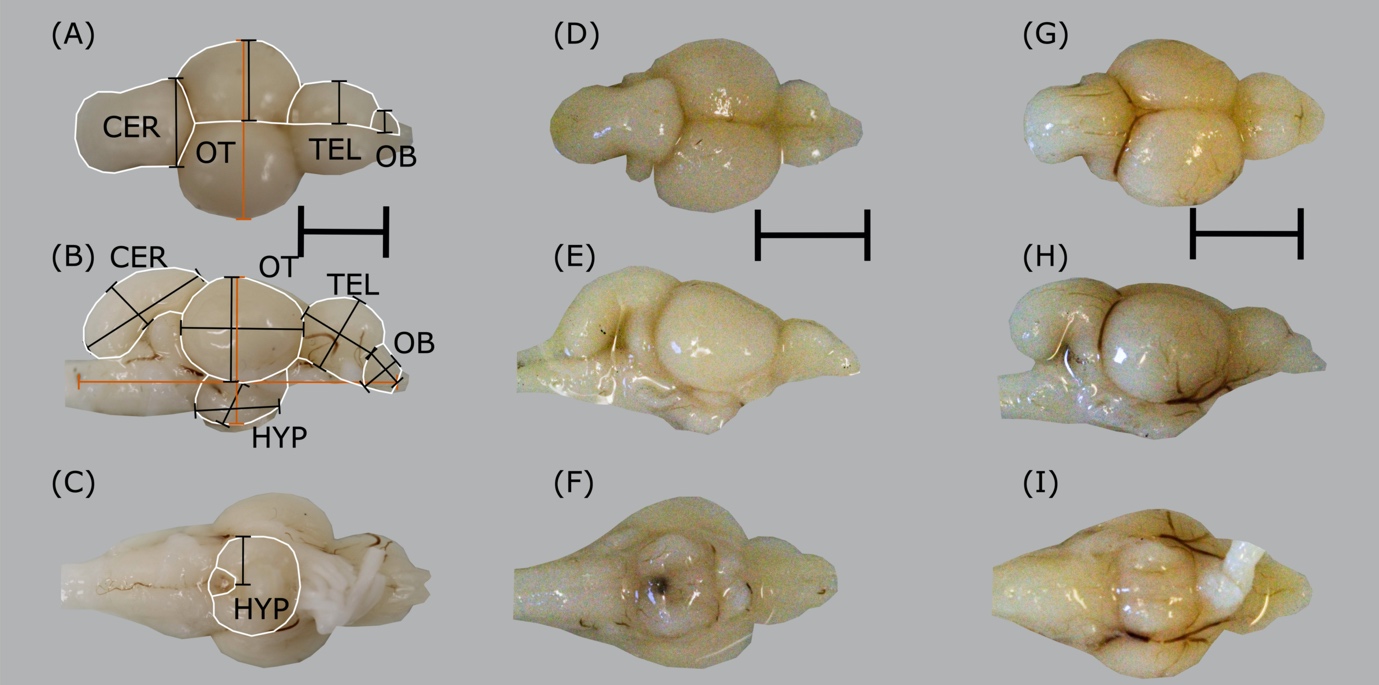
***

***Figure S1.1.*** – Dorsal/lateral/ventral view on brain of brown trout from lake (a-c), stream (d-f), and hatchery (g-i) environment. The thick black bar accompanying the dorsal view of brains is 5 mm scale. Outlines of the brain regions and measurements used for the volumetric analysis of each brain region are indicated by white and black lines respectively in figures a-c. The regions are from left to right and up to down order as follows: (a) dorsal view on cerebellum (CER), optic tectum (OT), telencephalon (TEL), olfactory bulb (OB), (b) lateral view on cerebellum, optic tectum, telencephalon, olfactory bulb, and hypothalamus (HYP), (c) ventral view on hypothalamus. The overall brain volume was estimated based on measurements indicated by the orange lines.

***Appendix 2 Morphometric analysis of body shape***

***Table S2.1.*** Pairwise comparisons of LS-means distances derived from Procrustes ANOVA (RRPP: 1,000 permutations) describing shape differentiation between brown trout from three habitat types

| **Pairwise comparison** | **Distance** | **UCL (95%)** | ***Z*** | ***p*** |
| --- | --- | --- | --- | --- |
| Lake – Stream | 0.0392 | 0.0560 | 9.787 | 0.001 |
| Lake – Hatchery | 0.0331 | 0.0138 | 9.367 | 0.001 |
| Stream – Hatchery | 0.0256 | 0.0142 | 6.388 | 0.001 |


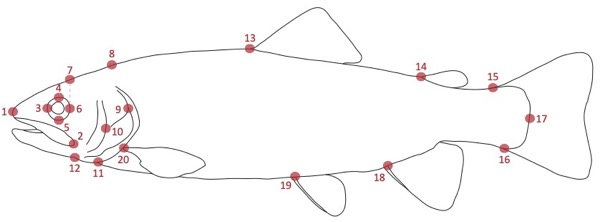


***Figure S2.1.*** Landmark placements for geometric morphometric analyses: (1) tip of snout; (2) posterior tip of maxilla; (3–6) anterior, superior, inferior and posterior of eye; (7) superior of cranium perpendicular to 6, indicated by broken line; (8) superior posterior of cranium; (9) anterior intersection of opercule and subopercule; (10) inferior intersection of opercule and preopercule; (11) ventral margin of opercula; (12) ventral margin of mandible; (13) anterior insertion of dorsal fin; (14) anterior insertion of adipose fin; (15–16) superior and inferior insertions of caudal fin; (17) junction of caudal fin at lateral line; (18) anterior insertion of anal fin; (19) anterior insertion of pelvic fin; (20) anterior insertion of pectoral fin.

***Figure S2.2.*** PCA plot of brown trout body shape, corrected for allometry (regressing Procrustes coordinates on centroid size) and lunate bending in PC2 (regressing coordinates on PC2 scores), and coloured by habitat type.

***Figure S2.3.*** Average body shapes of brown trout by habitat type (all exaggerated 2x for clarity).

***Appendix 3 – Results of the models considering only the subsample of individuals from the common-body-size window***

There was no significant difference in body size among trout from the three habitats within selected size window (*i.e.*, fork length <150, 215 > mm) (F_2,26_ = 1.89, p = 0.1718, model R^2^adj_._ = 0.060, Fig. S3.1a). The following results are based on the models considering only individuals from this body-size window. Overall brain volume was affected by the interaction between habitat and the fork length (FL) of individuals (F_2,23_ = 14.87, p < 0.001, model R^2^adj_._ = 0.846; Fig. S3.1a). The significant interaction indicates that, while there was a positive link between the overall brain volume and FL in wild populations *i.e.,* lake and stream habitat (F_1,12_ = 47.40, p < 0.001, model R^2^adj_._ = 0.898), hatchery fish showed no association between brain volume and FL (F_1,12_ = 1.5489, p = 0.2371, model R^2^adj_._ = 0.0405; Fig. Fig. S3.1a). Relative brain volume was larger in fish from lake than from stream habitat (F_1,12_ = 21.98, p = 0.001; Fig. S3.1a). The telencephalon volume increased with increasing volume of the whole brain (F_1,25_ = 17.20, p < 0.001, model R^2^adj_._ = 0.755, Fig. S3.1b); however; telencephalon volume also differed significantly between fish from different habitats (F_2,25_ = 7.648, p = 0.003). Specifically, the telencephala of stream dwelling trout were smaller than those of trout from both lake and hatchery environments (post-hoc p < 0.020), but the telencephala of lake and hatchery trout did not differ from each other (post-hoc p = 0.703). The volume of the optic tectum increased with the increasing volume of the whole brain (F_1,25_ = 16.43, p < 0.001, model R^2^adj_._ = 0.6912, Fig. S3.1c), but optic tectum volume did not differ significantly between fish from different habitats (F_2,25_ = 1.103, p = 0.347). Similarly, volumes of the olfactory bulb and cerebellum increased with the increasing volume of the whole brain (olfactory bulb: F_1,25_ = 10.63, p = 0.003, model R^2^adj_._ = 0.425, Fig. S3.1d; cerebellum: F_1,25_ = 13.14, p = 0.002, model R^2^adj_._ = 0.485, Fig. S3.1e), but volumes of these brain regions did not differ between individuals from different habitats (olfactory bulb: F_2,25_ = 0.730, p = 0.492; cerebellum: F_2,25_ = 0.378, p = 0.689). The volume of hypothalamus was not affected by brain size (F_1,25_ = 0.920, p =0.347; model R^2^adj_._ = 0.000) and it did not differ between the three environments (F_1,25_ = 0.171, p = 0.844, Fig. S3.1f).

***Figure S3.1*** – *The log-log scale relationship between a) overall brain volume and fork length (i.e., encephalization), and between overall brain volume and volume of b) the telencephalon, c) optic tectum, d) olfactory bulb, e) cerebellum, f) hypothalamus.*

***Table S3.1 –*** *Slope, Intercept, D.f. and R^2^_adj_ of linear models between FL and total brain volume, and (total brain volume – brain region volume) and the brain region volume by each habitat. The linear models are based on log transformed variables and correspond to the curves fitted in the Figure 3.1.*

| ***Habitat*** | ***Brain region*** | ***D.f.*** | ***Intercept*** | ***Slope*** | ***p-value*** | ***R^2^_adj_*** |
| --- | --- | --- | --- | --- | --- | --- |
| *Hatchery* | *Total brain* | 1;12 | -0.151 | -0.200 | 0.237 | 0.041 |
|  | *Telencephalon* | 1;12 | -3.607 | 0.498 | 0.367 | 0.000 |
|  | *Optic tectum* | 1;12 | -1.633 | 0.333 | 0.218 | 0.051 |
|  | *Olfactory bulb* | 1;12 | -4.617 | 1.170 | 0.305 | 0.011 |
|  | *Cerebellum* | 1;12 | -2.308 | 0.950 | 0.111 | 0.131 |
|  | *Hypothalamus* | 1;12 | -2.864 | 1.187 | 0.131 | 0.112 |
| *Stream* | *Total brain* | 1;5 | -6.274 | 0.991 | 0.010 | 0.715 |
|  | *Telencephalon* | 1;5 | -3.025 | 1.159 | 0.106 | 0.324 |
|  | *Optic tectum* | 1;5 | -0.587 | 0.990 | 0.008 | 0.741 |
|  | *Olfactory bulb* | 1;5 | -3.062 | 0.837 | 0.014 | 0.677 |
|  | *Cerebellum* | 1;5 | -2.519 | 0.822 | 0.100 | 0.338 |
|  | *Hypothalamus* | 1;5 | -4.093 | 0.523 | 0.716 | 0.000 |
| *Lake* | *Total brain* | 1;6 | -6.308 | 1.030 | 0.002 | 0.803 |
|  | *Telencephalon* | 1;6 | -2.326 | 1.554 | 0.010 | 0.640 |
|  | *Optic tectum* | 1;6 | -0.214 | 1.204 | 0.081 | 0.326 |
|  | *Olfactory bulb* | 1;6 | -4.909 | 0.857 | 0.267 | 0.066 |
|  | *Cerebellum* | 1;6 | -2.204 | 1.116 | 0.082 | 0.325 |
|  | *Hypothalamus* | 1;6 | -4.120 | 0.169 | 0.851 | 0.000 |
